# Arrb2 promotes endothelial progenitor cell-mediated postischemic neovascularization

**DOI:** 10.7150/thno.45133

**Published:** 2020-08-06

**Authors:** Xuelian Wang, Gaojian Huang, Jiaxin Mu, Zhilei Cong, Shuyan Chen, Dong Fu, Jia Qi, Zhen Li

**Affiliations:** 1 Department of Pharmacy, Xinhua Hospital, Shanghai Jiao Tong University School of Medicine, Shanghai, China.; 2 Department of Geriatrics, Xinhua Hospital, Shanghai Jiao Tong University School of Medicine, Shanghai, China.; 3 Department of Cardiology, Shanghai East Hospital of Clinical Medical College, Nanjing Medical University, Nanjing, China.; 4 Department of Emergency, Huashan Hospital affiliated to Fudan University, Shanghai, China.; 5 Department of General Surgery, Children's Hospital of Shanghai, Shanghai Jiao Tong University, Shanghai, China.

**Keywords:** angiogenesis, β-arrestin 2, endothelial progenitor cells, hind-limb ischemia, neovascularization

## Abstract

**Background and aim:** Modulating biological functions of endothelial progenitor cells (EPCs) is essential for therapeutic angiogenesis in ischemic vascular diseases. This study aimed to explore the role and molecular mechanisms of β-arrestin 2 (Arrb2) in EPCs biology and angiogenic therapy.

**Methods:** The influence of Arrb2 on postischemic neovascularization was evaluated in Arrb2-deficient mice. The proliferation, apoptosis, and various functions of EPCs were analyzed *in vitro* by manipulating the expression of Arrb2. Finally, the *in vivo* effect of Arrb2 on EPC-mediated neovascularization was investigated in a mouse model of hind-limb ischemia (HLI).

**Results:** Arrb2-deficient mice exhibited impaired blood flow recovery based on laser Doppler measurements and reduced capillary density in the adductor muscle after unilateral HLI. Arrb2-deficient mice also showed restricted intraplug angiogenesis in subcutaneously implanted Matrigel plugs. *In vitro*, lentivirus-mediated Arrb2 overexpression promoted EPC proliferation, migration, adhesion, and tube formation, whereas Arrb2 knockdown had opposite effects. In addition, the overexpression of Arrb2 in EPCs protected them from hypoxia-induced apoptosis and improved intraplug angiogenesis *ex vivo*. Mechanistically, Arrb2 interacted with and activated extracellular signal-regulated kinase (ERK)1/2 and protein kinase B (Akt) signaling pathways. Finally, the transplantation of EPCs overexpressing Arrb2 resulted in a significantly higher blood flow restoration in ischemic hind limb and higher capillary density during histological analysis compared with control or Arrb2-knockdown EPC-treated nude mice.

**Conclusions:** The data indicated that Arrb2 augmented EPC-mediated neovascularization through the activation of ERK and Akt signaling pathways. This novel biological function of Arrb2 might provide a potential therapeutic option to promote EPCs in the treatment of ischemic vascular diseases.

## Introduction

Postnatal neovascularization is critical to tissue repair and regeneration under various pathophysiological situations, such as wound healing, hypoxia, and ischemic vascular diseases 1, 2. Three different processes of vascular growth contribute to postischemic neovascularization in adults: angiogenesis, vasculogenesis and arteriogenesis 3, 4. Angiogenesis is defined as the sprouting of new blood vessels from preexisting vasculature 5. Arteriogenesis describes the growth and remodeling of a collateral artery 6. Vasculogenesis denotes the *de novo* formation of new vessels by angiogenic progenitor cells 7.

Endothelial progenitor cells (EPCs), first revealed two decades ago by Asahara and his colleagues in adult peripheral blood, are major players in vasculogenic processes 8, 9. Originated from the bone marrow and spleen, EPCs are preferentially recruited to ischemic sites and directly participate in vasculogenesis by adhesion, migration, proliferation, and endothelial differentiation 10, 11. EPCs also promote angiogenesis and arteriogenesis by eliciting paracrine effects 12, 13. Transplantation of EPCs has been demonstrated to enhance ischemic neovascularization and improve the perfusion and function of ischemic tissues in animal models of experimental ischemic injuries 14-16. Moreover, pilot clinical trials suggested that the therapeutic administration of EPCs was beneficial for patients with critical limb ischemia and acute myocardial infarction 17. However, the number and functional activity of EPCs was markedly impaired due to cardiovascular risk factors, such as diabetes, hypertension, hypercholesterolemia, and smoking 18, 19. The large-scale application of EPC-based therapy is also hindered by the poor viability of EPCs after transplantation 20. Therefore, new molecular approaches to modulate EPCs need to be explored to optimize the grafting efficacy of EPC therapy.

β-Arrestin 1 (Arrb1) and β-arrestin 2 (Arrb2) belong to the arrestin family and are abundantly expressed in the heart and vasculature 21, 22. They were initially defined as the crucial regulators of the G protein-coupling receptor 23. Accumulating studies suggested that Arrb2 might also serve as a signal transduction scaffold protein in various signaling pathways, including extracellular signal-regulated kinase (ERK) and protein kinase B (Akt) 24, 25. Recently, Arrb2 has been found to have an antiapoptotic effect in endothelial cells and induce their proliferation 26, 27. However, the roles of Arrb2 in vascular biology and its effects on EPCs have not been thoroughly studied. Therefore, the present study was performed to evaluate the influence of Arrb2 on postischemic neovascularization in Arrb2-deficient mice. Also, the proliferation, apoptosis, and various functions of EPCs were analyzed *in vitro* by manipulating the expression of Arrb2. Finally, the *in vivo* effect of Arrb2 on EPC-mediated neovascularization was investigated in a mouse model of hind-limb ischemia (HLI).

## Methods

### Mice

Arrb2 knockout (Arrb2^-/-^) mice (with a congenic C57BL/6 background) were generous gifts from Professor Gang Pei at the Institute of Biochemistry and Cell Biology, Shanghai Institutes for Biological Sciences, Chinese Academy of Sciences. Nude mice were bought from Shanghai SLAC Animal Company. All experiments were performed complying with the National Institutes of Health guidelines on the use of laboratory animals and approved by the ethics committee of the Xinhua Hospital affiliated to the Shanghai Jiao Tong University School of Medicine. The mice were sacrificed under anesthesia. All the mice were kept with ad libitum food and water in cages under a 12-h light/dark cycle.

### Late-outgrowth EPC culture and identification

EPCs were isolated from human umbilical cord blood and mouse bone marrow by density gradient centrifugation with Histopaque-1077/1083 (Sigma-Aldrich, MO, USA), suspended in complete endothelial cell growth medium-2 (EGM-2; Lonza, Switzerland), and then seeded in 12-well plates. They were characterized by labeling with Dil-conjugated acetylated low-density lipoprotein (Dil-ac-LDL; Invitrogen, USA) and flow cytometry. The cells were labeled with antibodies against CD34-Cy5.5, CD31-PE, VEGFR-APC, and CD45-APC (BioLengend, CA, USA) at 4°C for 30 min and washed twice with phosphate-buffered saline (PBS) before analysis with a FACSCanto II flow cytometer (BD, USA) following the manufacturer's protocols. Dual staining was used to examine the uptake of ac-LDL and the binding of fluorescein isothiocyanate (FITC)-conjugated UEA-1 (Sigma-Aldrich). EPCs were incubated with 10 μg/mL Dil-ac-LDL for 4 h at 37°C, fixed, washed, and incubated with 10 μg/mL FITC-UEA-1 at room temperature for 1 h.

### Lentiviral construct cloning, packaging, and viral infection

Lentivirus harboring β-Arrb2 sh-RNA (sh-Arrb2) or full-length Arrb2 expression vector (Lv-Arrb2) was generated and amplified as previously described with the help of GeneChem (Shanghai, China). EPCs were infected for 48 h (multiplicity of infection = 10), and the infection efficiency was confirmed by Western blot analysis. Pharmacological inhibitors of ERK1/2 (PD98059, No. HY-12028; MCE, NJ, USA) or Akt (MK-2206 2HCL, No.HY-10358; MCE) were added to EPC cultures 24 h after transfection.

### Hypoxia treatments

EPCs were starved in serum-free EGM-2 for 12 h before hypoxia treatment. Then, they were incubated with complete EGM-2 for 48 h in a sterilized hypoxic incubator with 94% N_2_ and 5% CO_2_ balanced with 1% O_2_.

### Proliferation assay

After 48 h transfection, EPCs were exposed to 20 μM 5-ethynyl-2'-deoxyuridine (Edu; RiboBio, China) for 2 h at 37 °C. Next, they were fixed, permeabilized, washed, and treated with 1× Apollo reaction cocktail for 30 min. Afterward, they were stained with DAPI (D1306, Thermo Fisher, USA) for 30 min. Ki67 staining was carried out following the manufacturer's protocols (ab16667; Abcam, USA). The EPCs proliferation (ratio of Edu+ or Ki67+ to all cells) was analyzed using images of randomly selected fields obtained on the fluorescence microscope (Leica, Germany).

### CCK-8 assay

The cell viability was measured using the CCK-8 (Dojindo, Japan) assay. After transfection with sh-Arrb2 or Lv-Arrb2 for 0, 24, and 48 h, CCK-8 solution (20 μL) and 180 μL of fresh culture medium were added to each well and incubated at 37°C for 2 h. The absorbance was read at 450 nm with a microplate reader (Bio-Rad, CA, USA). The relative cell viability was calculated as (OD450 of treated samples - OD450 of control samples)/(OD450 of untreated samples - OD450 of blank samples) × 100%.

### Flow cytometry analysis of the cell cycle

EPCs were collected 48 h after transfection, washed with ice-cold PBS, and fixed with 70% ethanol overnight at 4°C. The fixed cells were stained with 500 μL of PI/RNase staining buffer (550825, BD, USA) for 15 min at room temperature, followed by analysis with flow cytometry and ModFit Lt 4.1 software (Verity Software House, ME, USA).

### Scratch-wound assay

EPCs were transfected with sh-Arrb2 or Lv-Arrb2 for 48 h before the scratch assay. A scratch of the cell monolayer was made with a p200 pipette tip. The cells were cultured at 37°C for 12 h after washing with PBS and replacing it with a serum-free medium. EPCs were photographed at the time points of 0, 6, and 12 h, and Human Umbilical Vein Endothelial Cells (HUVEC) were photographed 0, 12, and 24 h after wounding. The closure area of the wound was calculated as follows: (*A*_0_ - *A*_n_)/*A*_0_ × 100%, where *A*_0_ represents the area of the initial wound and *A*_n_ represents the remaining area of the wound at that time point.

### Transwell assay

EPCs migration was also evaluated using the Transwell assay (Corning, NY, USA). Complete fresh EGM-2 (600 μL) was added to the bottom chambers. The cells transfected with lentivirus for 48 h were suspended in 100 μL of serum-free medium and added to the top chamber. After incubating at 37°C for 24 h, the membranes with migrated cells were fixed and then stained with crystal violet (Beyotime, China). The number of cells migrating into lower chambers were observed and counted under a microscope (Leica).

### Matrix adhesion assay

Adhesion assays were applied to evaluate cell adhesion ability. Equal cell numbers were seeded onto fibronectin-coated 6-well plates. After incubation at 37°C for 30 min, the cells were washed, fixed, stained with DAPI, and then counted in five randomly selected microscopic fields.

### Matrigel tube formation assay

Matrigel (10 μL; BD) was added to 96-well plates (ibidi, Germany) and incubated at 37°C for 30 min. Then, treated cells (2 ×10^4^/well) resuspended in 50 μL of complete EGM-2 were seeded on the Matrigel and incubated at 37°C. Tube formation was examined 6 h later. The digital images of endothelial tubes were photographed with a phase-contrast microscope (Olympus, Japan).

### *Ex vivo* Matrigel plug assay

The angiogenesis model was based on Matrigel (BD) implants in mice. Further, 500 μL of growth factor-reduced Matrigel with or without treated EPCs (5 × 10^5^) was injected subcutaneously into the middorsal region of 8-week-old male mice. The injected Matrigel rapidly formed a single, solid plug. After 2 weeks, the Matrigel plugs were surgically excised from the mice without connective tissues. The excised Matrigel was embedded into the paraffin and sectioned for immunofluorescence and immunohistochemistry.

### Western blot analysis

EPCs were washed and lysed in a buffer containing protease inhibitors and phosphatase inhibitors (Beyotime, China). Protein samples (20 μg) were loaded on 10% polyacrylamide gel, separated at 100 V for 90 min, and transferred at 100 V for 80 min. The PVDF membranes were blocked with QuickBlock sealing solution (Beyotime, China) for 15 min or 5% skimmed milk for 60 min at room temperature and incubated with primary antibodies against p-Akt (No. 9018, 1:1000, CST, MA, USA), Akt (No. 4691, 1:1000, CST), p-ERK1/2 (No. 4370, 1:1000, CST), ERK1/2 (No. 4695, 1:1000, CST), PP2A (No. 2290, 1:5000, CST), Arrb 1 (ab32099, 1:1000, Abcam), Arrb 2 (ab206972, 1:5000, Abcam), and GAPDH (No. 5174, 1:3000, CST) at 4°C overnight. Afterward, the membranes were subjected to secondary antibodies (1:3000, Jackson) at room temperature for 1 h. After washing with TBS-Tween (10 min, three times), the membranes were detected using a chemiluminescence detection system (Bio-Rad, CA, USA).

### Flow cytometry apoptosis assay

Cell apoptosis was analyzed by Annexin V-PE/7-AAD staining and flow cytometry (No. 559763, BD Biosciences, USA). The cells were collected, washed with PBS, resuspended in 200 μL of binding buffer, and then stained with 5 μL of Annexin V-PE and 5 μL of 7-AAD. Early apoptotic (PE positive, 7-AAD negative) and late apoptotic (PE positive, 7-AAD positive) cells were analyzed by flow cytometry.

### TUNEL staining

TUNEL staining was performed following the instruction of an In Situ Cell Death Fluorescein kit from Roche (No. 12156792910, Roche, Switzerland). In brief, the treated cells were incubated with 50 μL of TUNEL solution at 37°C for 1 h and photographed under a fluorescence microscope (Leica). The numbers of positive cells were quantified.

### Co-Immunoprecipitation

Co-Immunoprecipitation (Co-IP) was performed using a Pierce Co-Immunoprecipitation kit (Thermo Fisher). This method was mostly the same as in a previous study 28. Briefly, affinity-purified Arrb2 antibody (25 μg, ab206972, abcam) or Akt (25 μg, No. 4691, CST) was immobilized onto the resin for 90 min at room temperature. After washing with 1× coupling buffer three times, an equal amount of protein (500 μg) was added to the resin, and then, cells were incubated with gentle mixing overnight at 4°C. Finally, the protein was eluted and examined by Western blotting after washing four times with IP lysis buffer.

### HLI model

In brief, the left femoral artery was exposed, isolated from the femoral nerve and vein, and ligated at two positions 5 mm apart proximal to the groin ligament. Then, the segment of the femoral artery between the two ligations was completely cut with scissors 29. The vascular density in the injured adductor muscles was measured by CD31 immunofluorescence staining 21 days after the surgery.

### Micro-computed tomography

On postoperative day 21, micro-computed tomography (micro-CT) was also used to quantify and visualize the limb vasculature. Briefly, after anesthesia with pentobarbital (50 mg/kg i.p.), the right heart atrium was cut and the heart was perfused with heparinized saline, 4% paraformaldehyde, and Microfil working solution (42% MV-122, 53% diluent, and 5% curing agent) at a slow rate. Then, the mice were placed at 4°C for 2 h, and the legs were collected and fixed in 70% ethanol before micro-CT (Skyscan 1176, Bruker, Germany) scanning. Images were obtained after exposure to 50-kV, 500-μA, and at a resolution of 9-μm voxel size. Three-dimensional morphometric quantification was performed with a CT-Analyzer (Bruker).

### Laser Doppler imaging

Blood flow in hind limbs of mice was measured with Laser Doppler Imager (moor LDI2, Moor Instruments Ltd., UK) under anesthesia at different time points before and after the surgery. The images obtained were quantified with moor LDI Software Version 5.3. The limb perfusion ratio was calculated as the ischemic hind limb (IHL)/nonischemic hind limb (non-IHL) blood flow.

### Statistical analysis

Data were presented as mean ± standard deviation (SD). Differences between two groups were analyzed using the two-tailed paired *t* test. Differences among multiple groups were analyzed by two-way analysis of variance, followed by the Bonferroni's post-hoc test, a *p* <0.05 was considered statistically significant.

## Results

### Impaired ischemia-induced neovascularization in Arrb2-deficient mice

**Figure 1:**
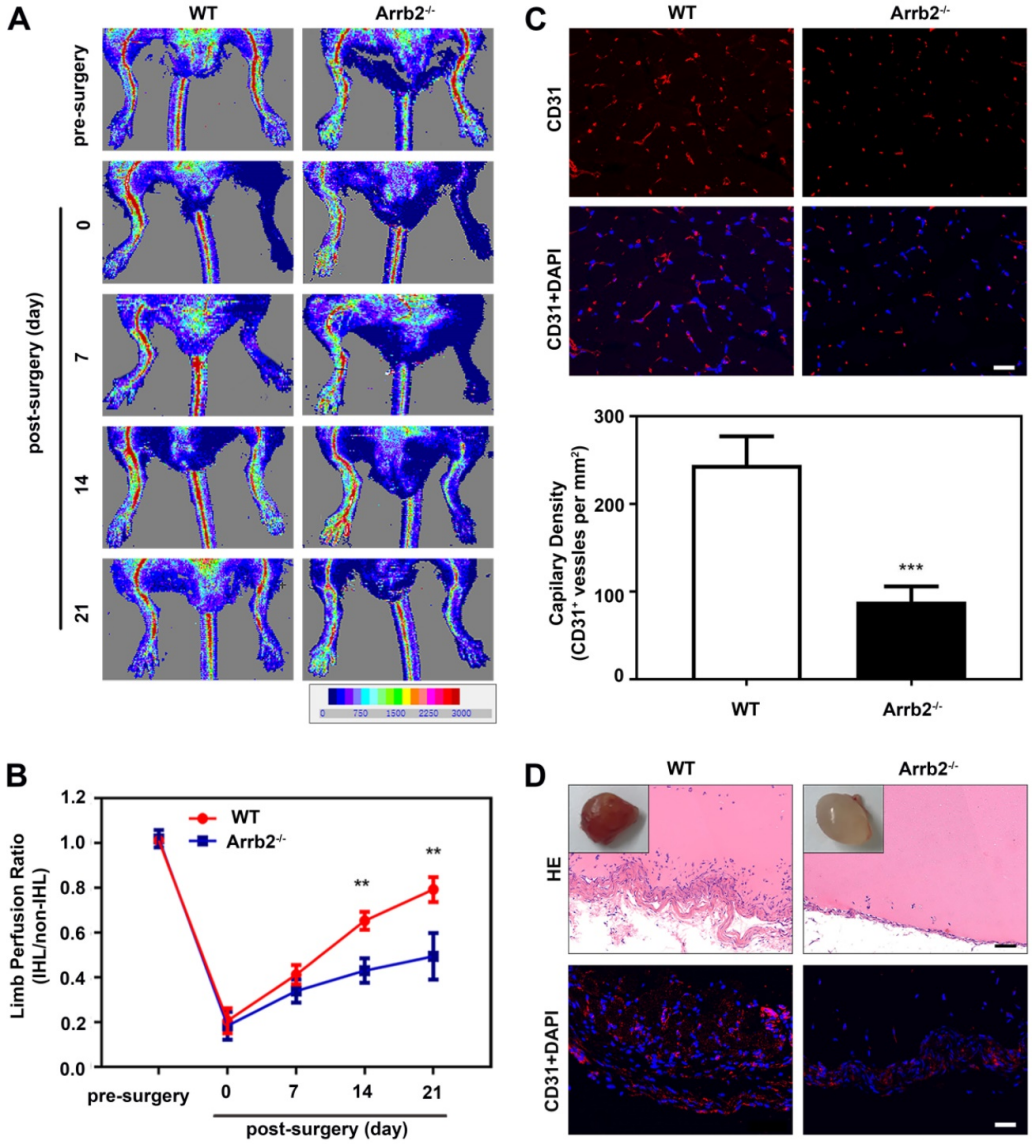
** Impaired ischemia-induced neovascularization in Arrb2-deficient mice.** Hind-limb ischemia (HLI) was surgically induced in WT and Arrb2^-/-^ mice. **(A and B)** Representative photographs from laser Doppler imaging (A) and quantified for each animal as the ratio of blood flow in the ischemic hind limb (IHL) and nonischemic hind limb (non-IHL) at different time points.** (C)** Representative photographs and quantification of anti-CD31 immunofluorescence staining of blood vessels 21 days after femoral artery ligation in WT and Arrb2^-/-^ mice. Scale bar = 50 µm. **(D)** Representative photographs of Matrigel plugs removed from WT and Arrb2^-/-^ mice 14 days after injection; HE and anti-CD31 staining of sections of Matrigel plugs removed from WT and Arrb2^-/-^ mice. Scale bar = 50 µm. All data were expressed as mean ± SD (*n* = 6). ^**^P < 0.01 compared with WT. ^***^P < 0.001 compared with WT.

A neovascularization model of HLI was used to evaluate the potential role of endothelial Arrb2 in neovascularization. The blood flow was monitored over time by laser Doppler imaging. Laser Doppler analyses displayed a similar degree of reduced blood flow in ligated limbs of Arrb2^-/-^ mice and wild-type (WT) littermates after the surgery compared with respective nonligated contralateral limbs, indicating comparable postoperative ischemia in both strains. In WT mice, blood flow recovered to almost baseline levels by 21 days after the surgery. However, blood flow recovery was significantly impaired in Arrb2^-/-^ mice (Figure 1A and B). Similarly, lower anti-CD31-positive capillary density was observed in the Arrb2^-/-^ histology of the adductor muscle than in WT mice after 21 days (Figure 1C). Besides, the Matrigel plug assay was used to explore further the potential function of Arrb2 in angiogenesis *ex vivo*. On gross examination, Matrigel plugs from WT mice were reddish brown. However, plugs from Arrb2^-/-^ mice were paler (Figure 1D). Blood vessel infiltration in implants was also quantified by immunostaining with an anti-CD31 antibody. Compared with Matrigel-implanted WT mice, Arrb2^-/-^ mice showed decreased vascularization in plugs (Figure 1D). Therefore, Arrb2 might contribute to revascularization after femoral artery ligation-induced ischemia.

### Arrb2 increased EPCs proliferation and alleviated apoptosis

Given that EPCs is an important regulator of vascularization, this study investigated the function of Arrb2 in EPCs biology. EPCs were isolated from human cord blood and mouse bone marrow by density gradient centrifugation. After 15-day culture of mononuclear cells, spindle-shaped or cobblestone-like adherent cells were observed (Figure S1A). These cells were able to take up Dil-ac-LDL and bind to FITC-UEA-1, which were characteristic functions of endothelial cells (Figure S1B). Moreover, these cells also expressed progenitor cell-specific surface antigen CD34 and endothelial cell-specific surface antigens VEGFR2 and CD31 (Figure S1C). Therefore, these cells were confirmed as EPCs.

This study first investigated the expression of Arrb1 and Arrb2 in EPCs after HLI induction. As shown in Figure S2A, the protein expression level of Arrb2 profoundly reduced compared with that in the nonischemia group. Similarly, the Arrb2 expression level overtly decreased in EPCs after hypoxic treatment (Figure S2B). On the contrary, neither HLI nor hypoxia injury altered the expression of Arrb1 at the protein level in EPCs (Figure S2). These results suggested Arrb2, not Arrb1, might be the Arrb isoform involved in EPC-mediated postischemic neovascularization.

**Figure 2:**
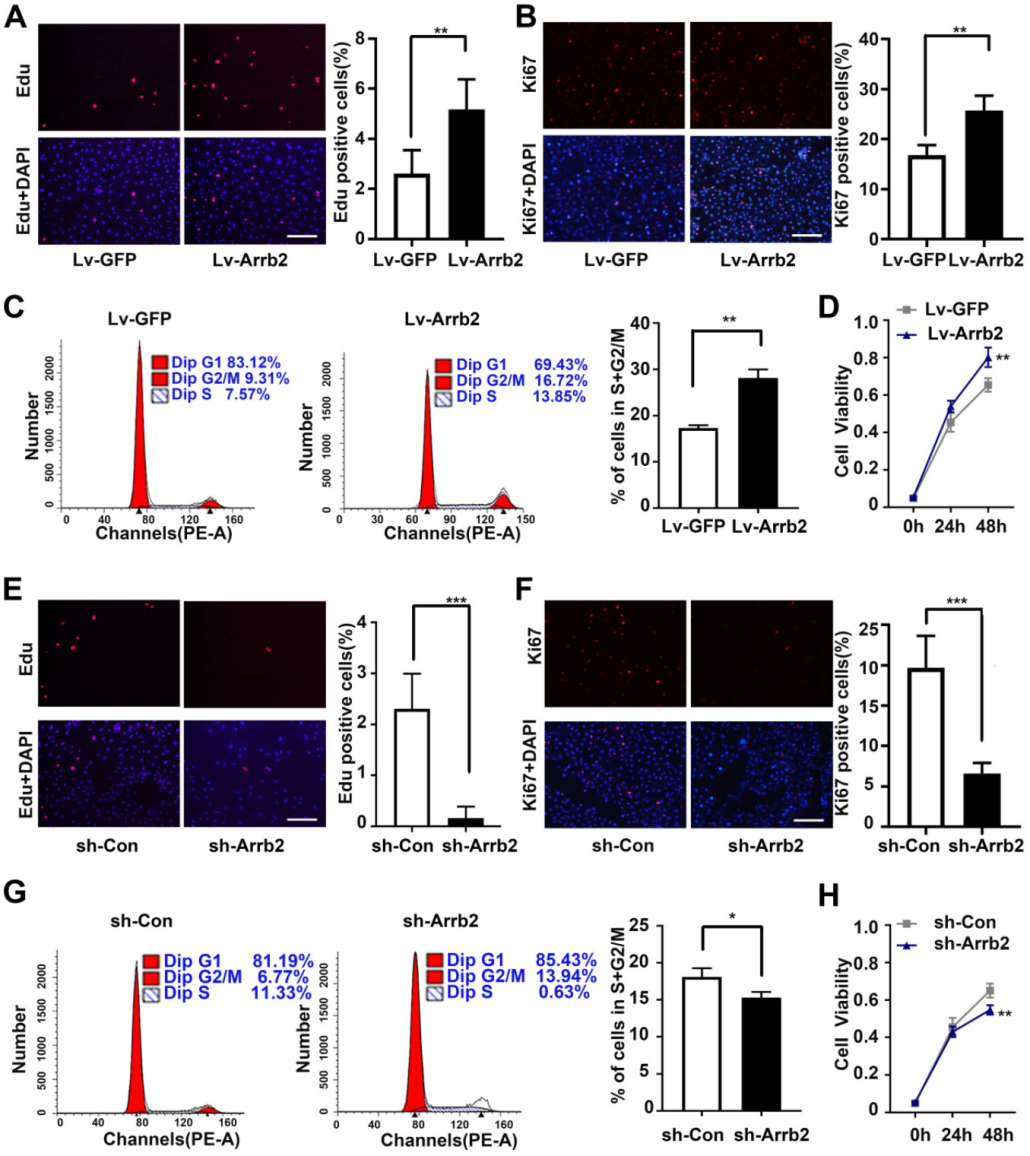
** Effects of Arrb2 on EPC proliferation. (A and B)** Representative images and quantification of Edu staining (A) and Ki67 staining (B) showing the effects of Lv-Arrb2 on the proliferation of EPCs. Scale bar = 200 µm. **(C)** Cell cycle distribution was analyzed by flow cytometry, and the percentage of cells in the S + G2/M phase was quantified to examine the effect of Lv-Arrb2 on proliferation.** (D)** The cell counting kit assay was used to show the effect of Lv-Arrb2 on viability. **(E and F)** Representative images and quantification of Edu staining (E) and Ki67 staining (F) to show the effects of sh-Arrb2 on the proliferation of EPCs. Scale bar = 200 µm.** (G)** Cell cycle distribution was analyzed by flow cytometry, and the percentage of cells in S + G2/M phase was quantified to examine the effect of sh-arrb2 on proliferation. **(H)** The cell counting kit assay was used to present the effect of sh-Arrb2 on viability. All data were expressed as mean ± SD (*n* = 6). ^*^P < 0.05, ^**^P < 0.01, ^***^P < 0.001 compared with the control group.

**Figure 3:**
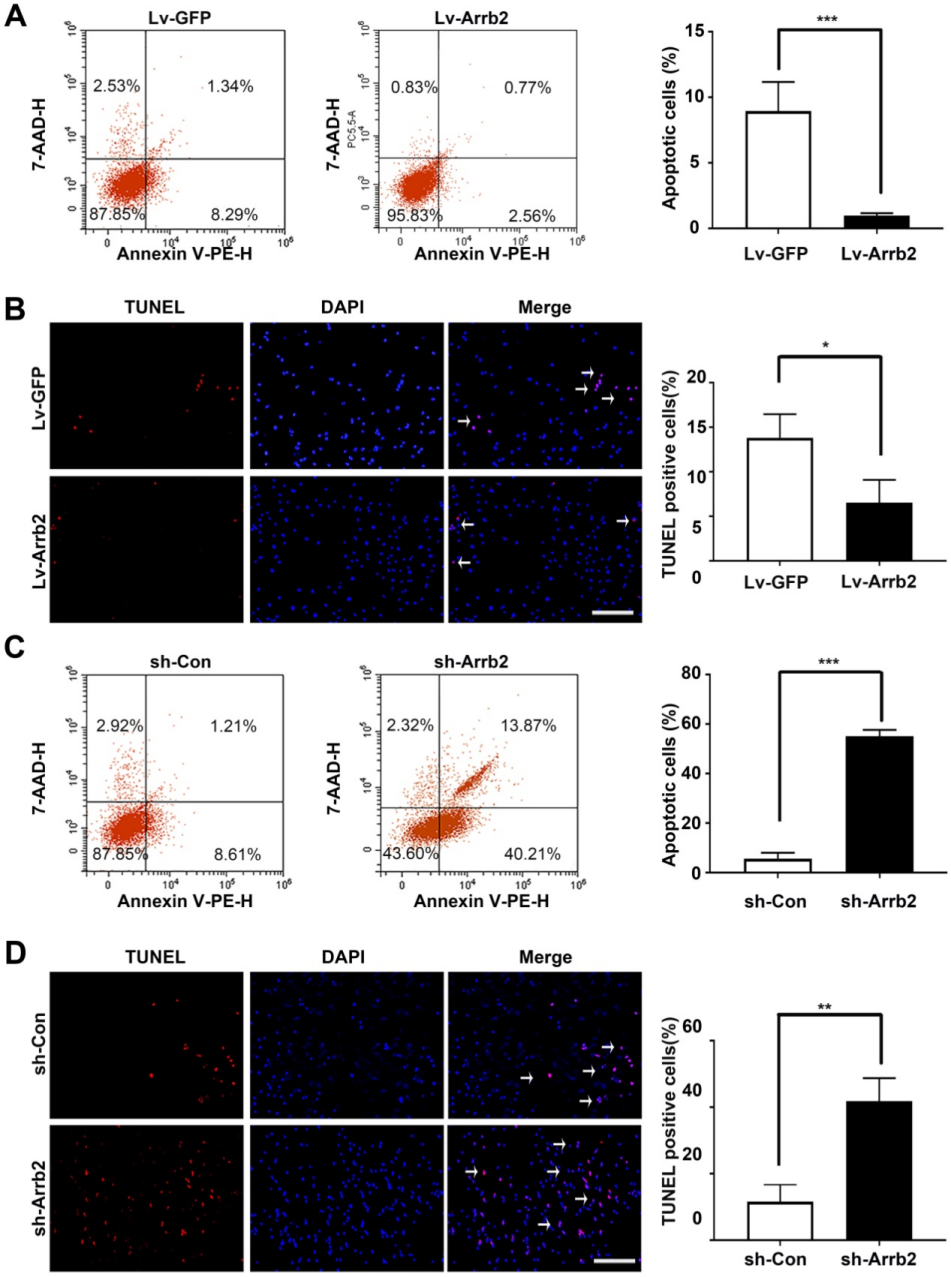
** Effects of Arrb2 on EPC apoptosis. (A)** The cells were transfected with Lv-Arrb2, and cell apoptosis was analyzed by flow cytometry 48 h later. The percentage of apoptotic cells was quantified to examine the effect of Lv-Arrb2 on EPC apoptosis.** (B)** Representative images of TUNEL staining in EPCs and quantification of TUNEL-positive cells (white arrows) to evaluate the effect of Lv-Arrb2 on cell apoptosis. Scale bar = 200 µm. **(C)** The cells were transfected with sh-Arrb2, and cell apoptosis was analyzed by flow cytometry 48 h later. The percentage of apoptotic cells was quantified to examine the effect of sh-Arrb2 on EPC apoptosis. **(D)** Representative images of TUNEL staining in EPCs and quantification of TUNEL-positive cells (white arrows) to evaluate the effect of sh-Arrb2 on cell apoptosis. Scale bar = 200 µm. All data were expressed as mean ± SD (*n* = 5). ^*^P < 0.05, ^**^P < 0.01, ^***^P < 0.001 compared with the control group.

Next, the study assessed the contribution of Arrb2 to the biological functions of EPCs using gain- and loss-of-function approaches. The efficiency of lentivirus-mediated overexpression and knockdown of Arrb2 was determined by Western blot analysis (Figure S3). As shown in Figure 2A-D, the number of Edu and Ki67-positive cells, number of cells in the S + G2/M phase, and cell viability significantly increased in the Lv-Arrb2 treatment group. The opposite effects were observed in the group treated with sh-Arrb2 (Figure 2E-H), indicating that Arrb2 promoted EPCs proliferation. In addition, flow cytometry analysis and TUNEL assay showed that Arrb2 overexpression protected EPCs from hypoxia-induced apoptosis (Figure 3A and B), while the knockdown of Arrb2 in EPCs aggravated cell apoptosis (Figure 3C and D).

### Arrb2 improved EPCs migration and adhesion

**Figure 4:**
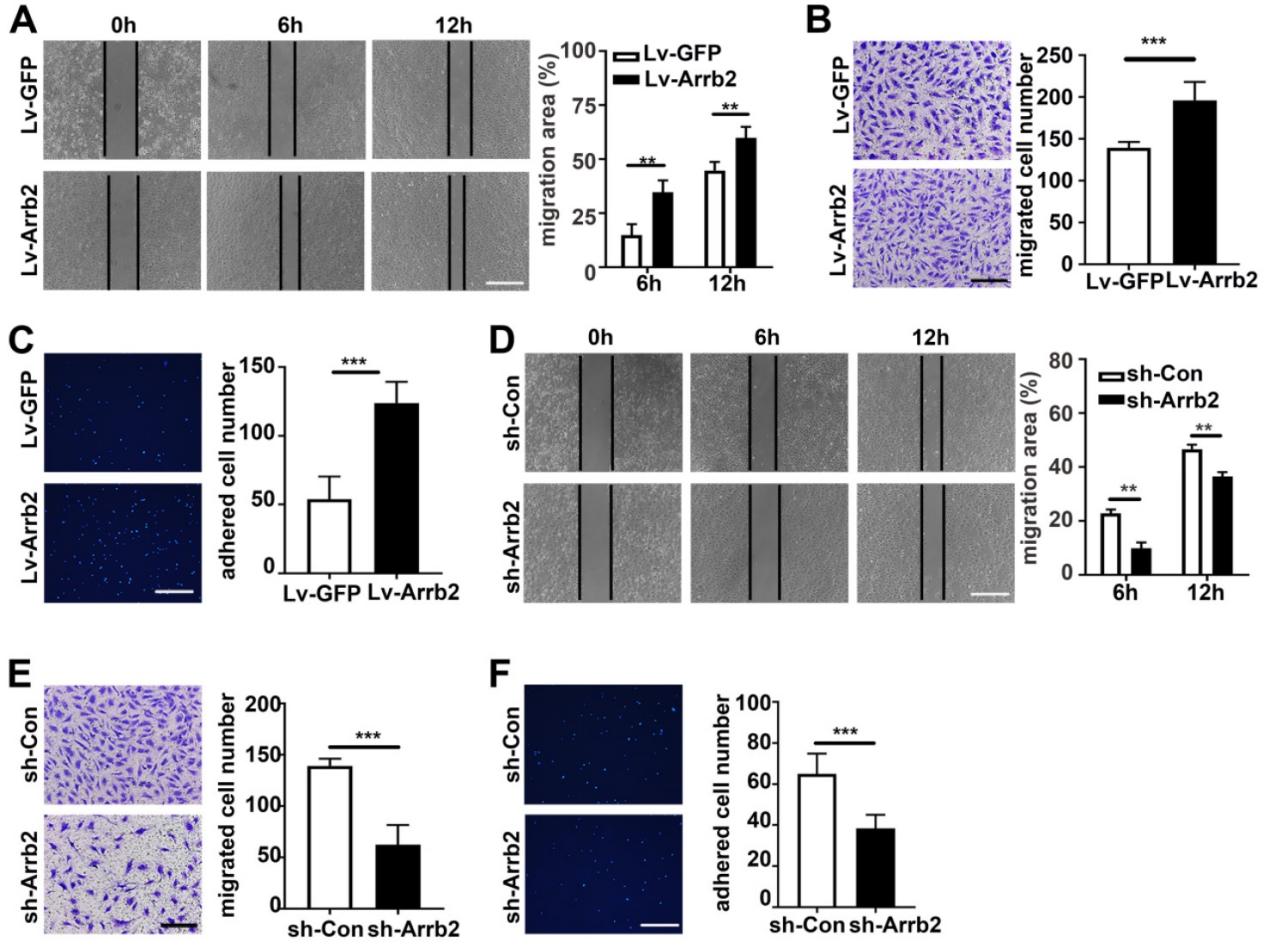
** Arrb2 improved EPC migration and adhesion. (A-C)** EPCs were infected with Lv-Arrb2 or Lv-GFP for 48 h. Representative images of the *in vitro* scratch-wound assay and quantification of the migration area (A) were presented to show the effects of Lv-Arrb2 on cell migration. Scale bar = 400 µm. Representative images of the Transwell migration assay and quantification of the migrated cells (B) were presented to show the effects of Lv-Arrb2 on cell migration. Scale bar = 200 µm. Representative images and quantification of adhered cells (C) were presented to show the effects of Lv-Arrb2 on cell adhesion. Scale bar = 400 µm. **(D-F)** EPCs were infected with sh-Arrb2 or sh-Con for 48 h. Representative images of the *in vitro* scratch-wound assay and quantification of the migration area (D) were presented to show the effects of sh-Arrb2 on cell migration. Scale bar = 400 µm. Representative images of the Transwell migration assay and quantification of the migrated cells (E) were presented to show the effects of sh-Arrb2 on cell migration. Scale bar = 200 µm. Representative images and quantification of adhered cells (F) showing the effects of sh-Arrb2 on cell adhesion. Scale bar = 400 µm. All data were expressed as mean ± SD (*n* = 5). ^**^P < 0.01, ^***^P < 0.001 compared with the control group.

The *in vitro* scratch-wound and Transwell migration assays were used to evaluate the effect of Arrb2 on EPCs migration. As expected, the *in vitro* scratch-wound assay showed that cell migration was significantly stimulated by Lv-Arrb2 and inhibited by sh-Arrb2 (Figure 4A and 4D). Similar results were observed in the Transwell migration assay (Figure 4B and 4E). Moreover, Arrb2 overexpression promoted, while Arrb2 knockdown inhibited, endothelial cells migration (Figure S4).

EPCs were pretreated with Lv-Arrb2 or sh-Arrb2 for 48 h, seeded on collagen type I and fibronectin-coated plates, and incubated for 1 h at 37°C to examine the effect of Arrb2 on EPCs adhesiveness. The number of adhesive EPCs significantly increased in the Lv-Arrb2 treatment group but decreased in the sh-Arrb2 treatment group compared with the control group (Figure 4C and 4F).

### Effects of Arrb2 on angiogenic function

**Figure 5:**
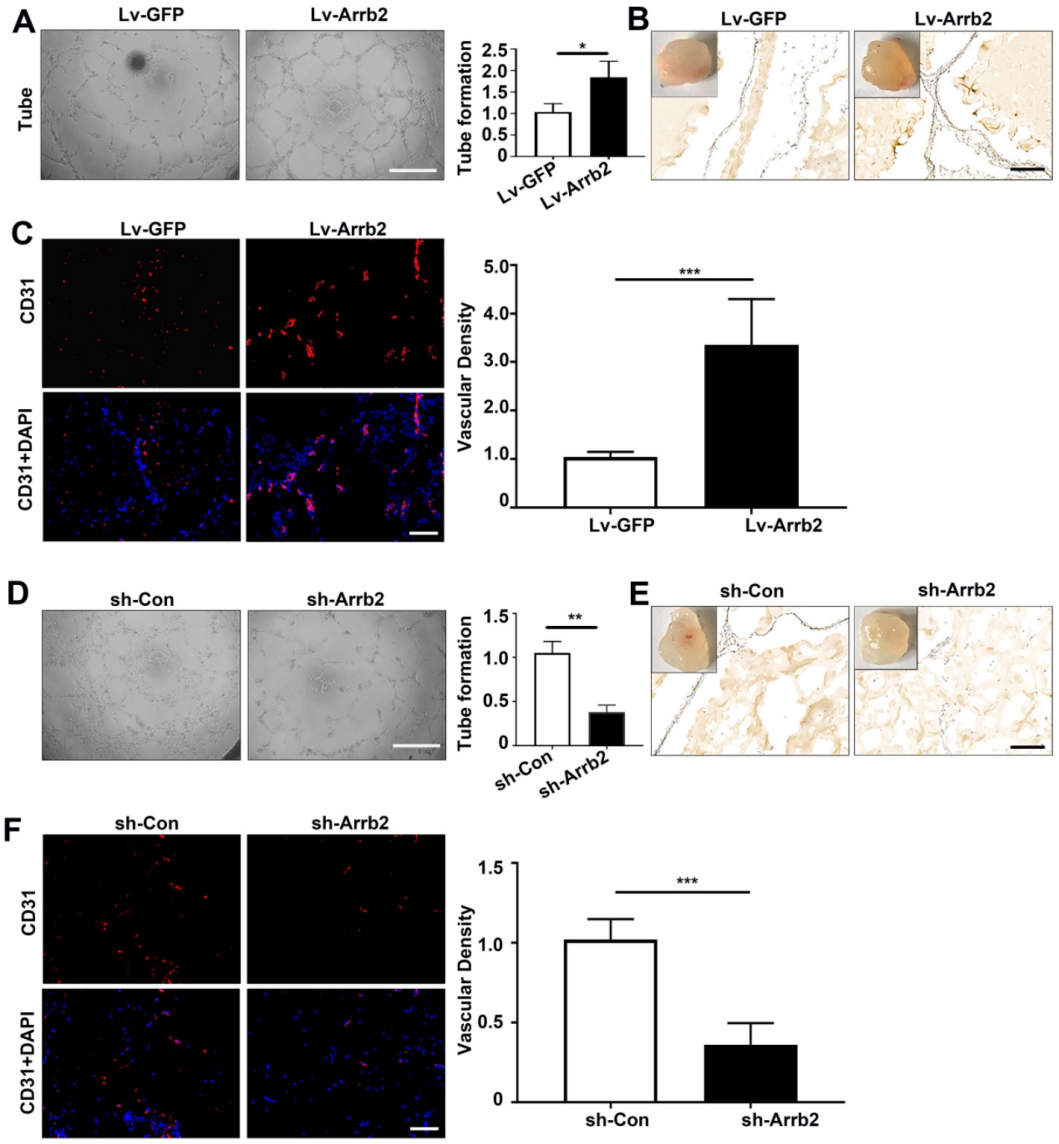
** Effects of Arrb2 on angiogenic function. (A-C)** EPCs were infected with Lv-Arrb2 or Lv-GFP for 48 h. Representative images and quantification of tube formation (A) showing the effects of Lv-Arrb2 on angiogenic function *in vitro*. Scale bar = 400 µm. Representative images and quantification of immunohistochemistry (B) and anti-CD31 staining (C) of sections of Matrigel plugs were presented to show the effects of Lv-Arrb2 on EPCs angiogenesis *ex vivo*. Scale bar = 200 µm. **(D-F)** EPCs were infected with sh-Arrb2 or sh-Con for 48 h. Representative images of tube formation (D) showing the effects of sh-Arrb2 on angiogenic function *in vitro*. Scale bar = 400 µm. Representative images and quantification of immunohistochemistry (E) and anti-CD31 (F) staining of sections of Matrigel plugs showing the effects of sh-Arrb2 on EPCs angiogenesis *ex vivo*. Scale bar = 200 µm. All data were expressed as mean ± SD (*n* = 5). ^*^P < 0.05, ^**^P < 0.01, ^***^P < 0.001 compared with the control group.

The tube formation assay was used to evaluate the effects of Arrb2 on angiogenic function. Tube formation significantly increased in Lv-Arrb2-treated cells compared with the control group. However, tube formation was significantly inhibited in sh-Arrb2-treated cells (Figure 5A and 5D). Furthermore, the Matrigel plug assay was performed with EPCs transfected with Lv-Arrb2 or sh-Arrb2 to test the effects of Arrb2 on EPCs angiogenesis *ex vivo*. In line with the results observed in the tube formation assay, Matrigel containing EPCs overexpressing Arrb2 displayed significantly improved neovascularization compared with the control group (Figure 5B and C). In contrast, plugs containing EPCs silencing Arrb2 showed a marked reduction in neovascularization compared with the control group (Figure 5E and F).

### Upregulating Arrb2 expression activated ERK and Akt in EPCs

**Figure 6:**
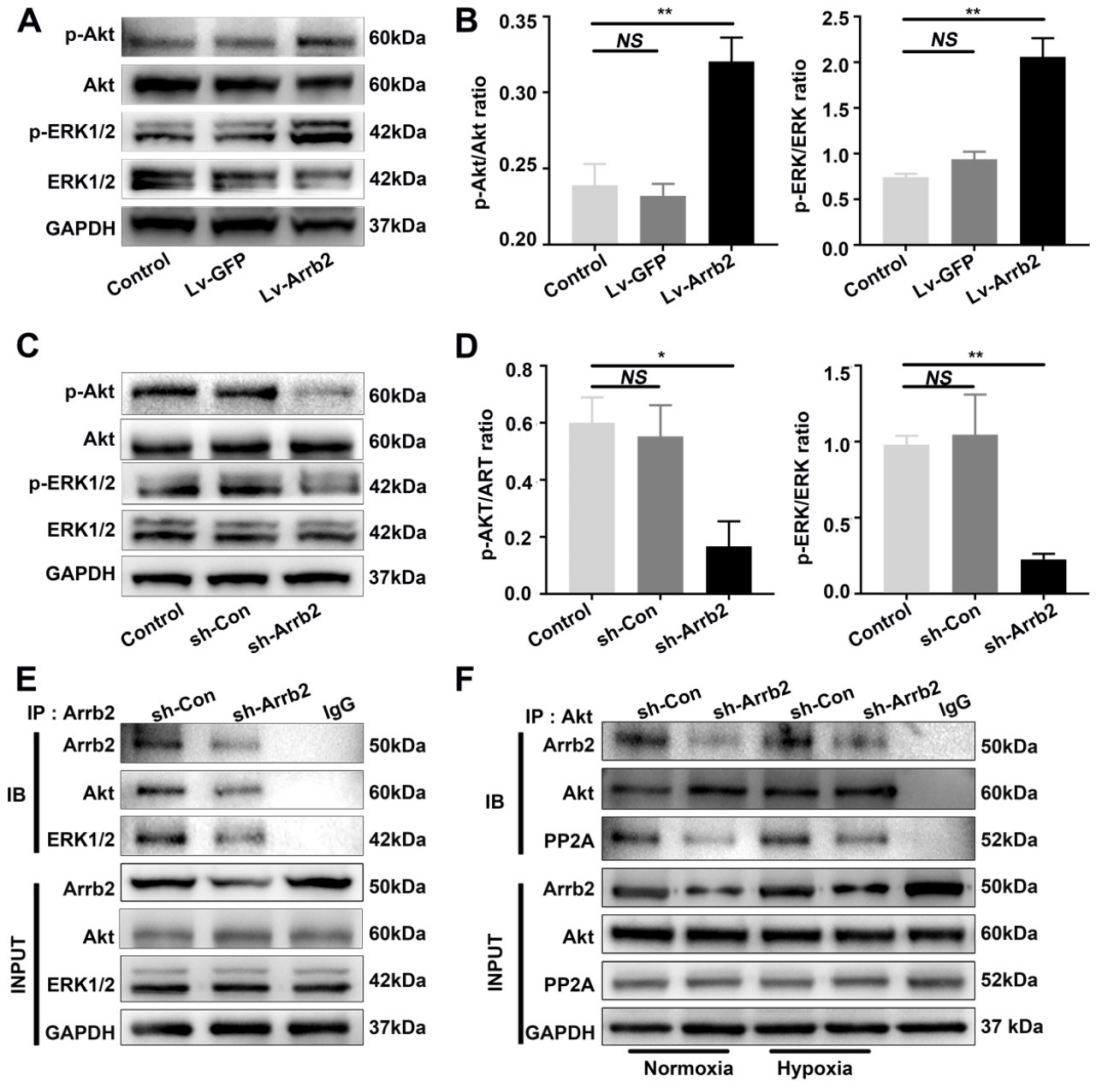
** Effects of Arrb2 on ERK and Akt activation in EPCs. (A and B)** EPCs were infected with Lv-Arrb2 or Lv-GFP for 48 h. The effects of Lv-Arrb2 on ERK1/2 and Akt phosphorylation were determined by Western blot analysis (A) and then quantitated by densitometric analysis (B).** (C and D)** EPCs were infected with sh-Arrb2 or sh-Con for 48 h. The effects of sh-Arrb2 on ERK1/2 and Akt phosphorylation were determined by Western blot analysis (C) and then quantitated by densitometric analysis (D). **(E)** Protein expression of ERK1/2 and Akt precipitated by Arrb2 from EPCs was examined by Western blot analysis.** (F)** Protein expression of Akt and PP2A precipitated by Akt from EPCs under normoxic and hypoxic conditions was examined by Western blot analysis. All data were expressed as mean ± SD (*n* = 3). ^*^P < 0.05, ^**^P < 0.01 compared with the control group.

As Arrb2 reportedly serves as a signal transduction scaffold protein in ERK and Akt signaling pathways, this study investigated the effects of Arrb2 on the activation of ERK and Akt in EPCs. In agreement with other cell types, both ERK1/2 and Akt were significantly activated by Lv-Arrb2 treatment but inhibited by sh-Arrb2 (Figure 6A-D). The interaction between ERK1/2 or Akt and Arrb2 was confirmed by the Co-IP assay (Figure 6E). Furthermore, the decrease in proliferation, migration, and tube formation in Arrb2-knockdown EPCs was aggravated by the pharmacological inhibitors of ERK1/2 (PD98059; Figure S5) and Akt (MK-2206 2HCL; Figure S6), suggesting that Arrb2 controlled EPC function through ERK1/2 and Akt pathways.

Previous studies showed that PP2A was also involved in the regulation of Akt signaling by Arrb2. The protein level of the PP2A C subunit (the catalytic subunit), which was the major subunit interacting with Arrb2, was not changed by Arrb2 knockdown (Figure 6F). Moreover, the interaction between PP2A and Akt was abolished by Arrb2 knockdown in EPCs, but the formation of PP2A/Akt complex was not affected by hypoxic treatment (Figure 6F), indicating that PP2A was unlikely involved in Arrb2-induced activation of Akt signaling in EPCs under hypoxia conditions.

### Arrb2 stimulated EPCs-mediated neovascularization *in vivo*

**Figure 7:**
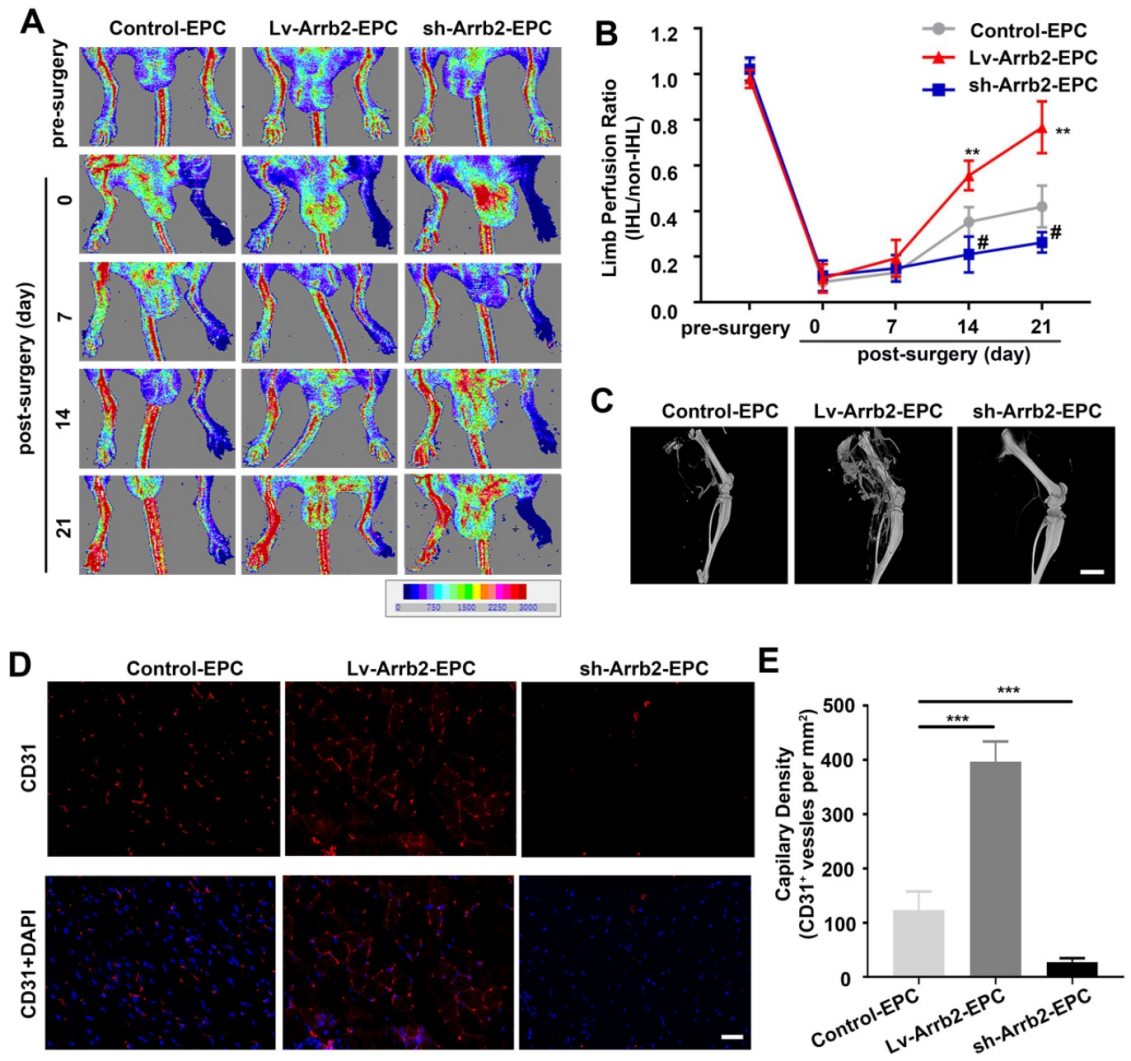
** Arrb2 contributed to EPC-mediated neovascularization *in vivo*.** Hind-limb ischemia (HLI) was surgically induced in 8-week-old nude mice. Immediately after HLI induction, the nude mice were injected with noninfected EPCs (Control) or EPCs infected with Lv-Arrb2/sh-Arrb2. **(A and B)** On days 0, 7, 14, and 21 after HLI, blood flow was evaluated using laser Doppler imaging (A) and quantified for each mice at different time points (B).** (C)** Representative images of 3D reconstructions of the femoral bone and blood vessels of mice on day 21 after the surgery. Scale bar = 500 mm. **(D and E)** 21 days after HLI, the adductor muscle was harvested from the HLI limb and stained for CD31 expression (D). Capillary density (E) was calculated as the number of CD31^+^ vessels per mm^2^. Scale bar = 50 µm. All data were expressed as mean ± SD (*n* = 5), ^#^*P* < 0.05, ^**^P < 0.01, ^***^P < 0.001 compared with the control group.

**Figure 8:**
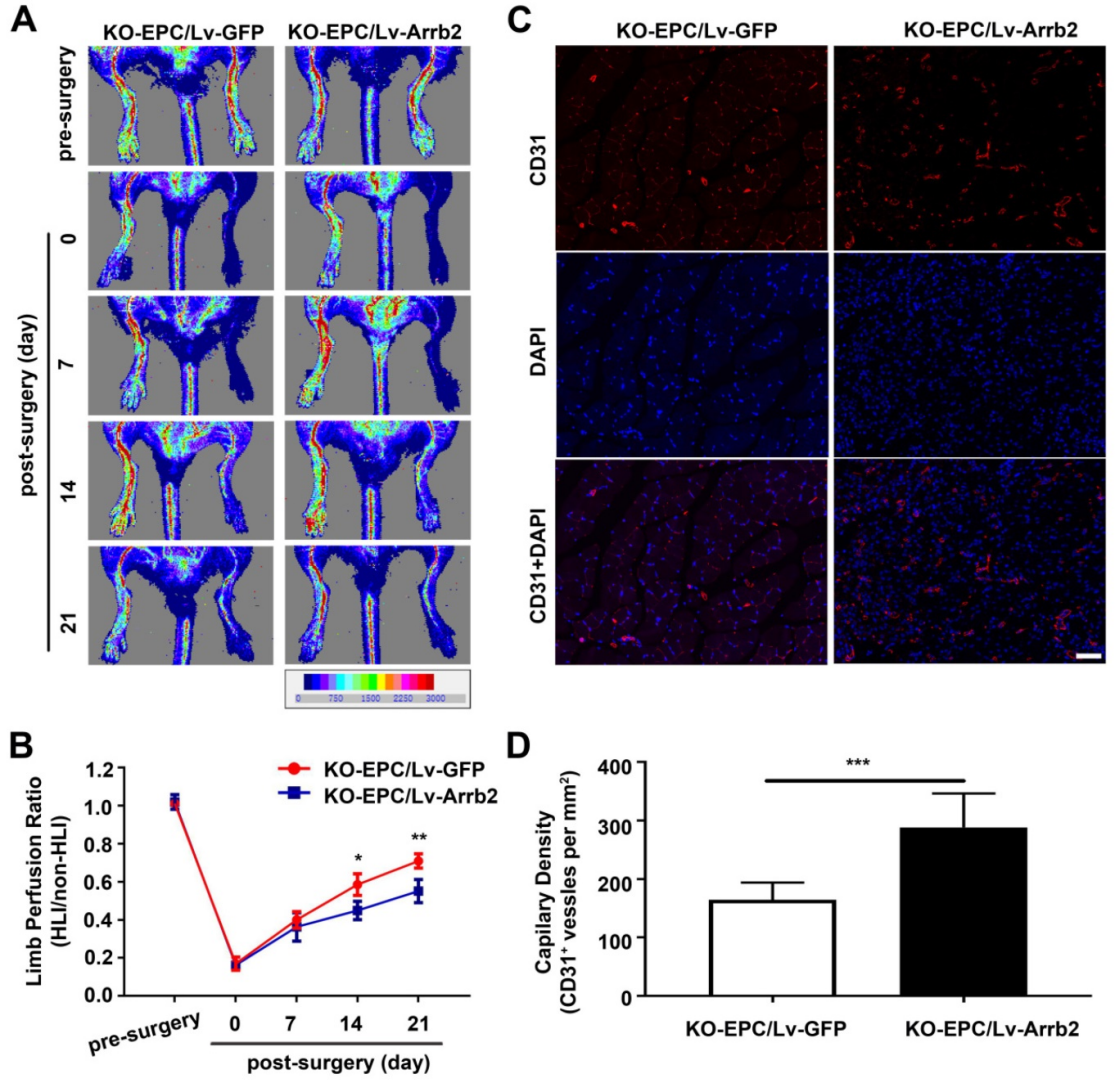
** Arrb2 overexpression rescued EPC-mediated neovascularization in the ischemic limb of Arrb2^-/-^ mice.** EPCs isolated from Arrb2^-/-^ mice bone marrow (KO-EPC) were transfected with Lv-Arrb2 or Lv-GFP and injected into 8-week-old Arrb2^-/-^ mice after unilateral hind-limb ischemia (HLI) surgery.** (A and B)** Time course of blood perfusion shown in images (A) and quantitative data analysis (B) after transplantation of transfected KO-EPCs. Blood perfusion is the ratio of ischemic to nonischemic limb perfusion measured using moor LDI software. **(C and D)** Immunofluorescent staining (C) and quantitation (D) of CD31-positive capillaries in transverse sections of adductor muscle tissue from ischemic hind limbs 21 days after HLI surgery. Capillary density was expressed as CD31-positive capillaries per mm^2^. Scale bar = 50 µm. All data were expressed as mean ± SD (*n* = 5), ^*^P < 0.05, ^**^P < 0.01, ^***^P < 0.001 compared with the control group.

EPCs infected with Lv-Arrb2 or sh-Arrb2 were injected immediately after HLI induction in nude mice to determine whether Arrb2 regulated angiogenesis after ischemic injury. Laser Doppler perfusion imaging analysis was performed on days 0, 7, 14, and 21 after ischemia to evaluate limb perfusion ratio (IHL/non-IHL). Laser Doppler analyses displayed a similar degree of reduced blood flow in ligated limbs of mice after surgery compared with respective nonligated contralateral limbs, indicating comparable postoperative ischemia in both strains. Reperfusion in the IHL improved when EPCs infected with Lv-Arrb2 were injected, compared with control EPCs. However, blood flow recovery was significantly impaired in the sh-Arrb2-EPCs-treated group (Figure 7A and B). Similar results were also observed by micro-CT 21 days after the surgery (Figure 7C). Moreover, immunofluorescence showed that higher anti-CD31-positive capillary density was observed in the immunofluorescence of the adductor muscle in the Lv-Arrb2-EPCs-treated group than in control mice after 21 days, but it was lower in the sh-Arrb2-EPCs-treated group (Figure 7D and E). In addition, EPCs isolated from Arrb2^-/-^ mice (KO-EPCs) were transfected with Lv-Arrb2 and reinjected into the tail vein of Arrb2^-/-^ mice (Figure S7). As shown in Figure 8, blood flow recovery and capillary density were rescued in Arrb2^-/-^ mice after transplantation of Lv-Arrb2-treated KO-EPCs.

## Discussion

The present study was novel in demonstrating that a deficiency of Arrb2 resulted in impaired neovascularization following HLI. It also showed that the knockdown of Arrb2 in EPCs impaired their survival and function, whereas Arrb2 overexpression enhanced their angiogenic activity both *in vitro* and *in vivo*. The mechanisms involved were consistent with the activation of ERK and Akt signaling pathways. Collectively, these findings suggested a new biological role of Arrb2 in neovascularization and a novel therapeutic intervention to ischemic vascular disease.

Arrb2 is a ubiquitously expressed multifunctional protein by regulating G protein-coupled receptor desensitization and internalization 23, 30. Previous studies using Arrb2 knockout animals provided evidence for the specific mediation of biological effects by Arrb2 21, 31. In line with these findings, the results of the present study showed that blood flow recovery and capillary formation following HLI were substantially impaired in Arrb2^-/-^ mice. Limb ischemia, as a consequence of vascular injury, initiates a series of events involving cell infiltration and neovascularization 32. EPCs, a subtype of stem cells with high proliferative potential, are capable of differentiating into mature endothelial cells and have been proved as an important contributor to neovascularization 12, 33. Therefore, this study investigated the effects of Arrb2 in EPCs and found that Arrb2 could increase EPCs proliferation and alleviate apoptosis. Moreover, Arrb2 improved EPCs migration, adhesion, and angiogenic function *in vitro* and *ex vivo*.

The discovery of EPC has resulted in a paradigm shift in vascular biology, indicating that adult vessels can be repaired not exclusively by local endothelial cells proliferation, migration, and remodeling, but also by incorporation of EPCs in sprouting new blood vessels 17, 34. Several studies demonstrated that EPCs were recruited to the site of vascular damage where they migrated, proliferated, and adhered to the vessel wall, promoting the reendothelialization of damaged vessels and inducing vasculogenesis in the ischemic areas 35, 36. The present study showed that Arrb2 was sequentially involved in different steps of this process.

Arrb2 can induce cell proliferation and promote cell migration in other tissues. Kim et al. recently demonstrated that Arrb2 induced smooth muscle cell proliferation and migration through ERK- and Akt-dependent mechanisms 24. Previous studies showed that the ERK1/2 signaling pathway promoted cell survival through the inactivation of components of cell death machinery and enhancement of the transcription of prosurvival genes 37, 38. In addition, different studies demonstrated that Akt acted as an integrator of multiple signal transduction pathways, controlled many critical steps in angiogenesis, such as cell migration and vascular network formation, and promoted cell survival by inhibiting apoptosis 11, 39. The present study demonstrated that Arrb2 interacted and activated ERK1/2 and Akt in EPCs. The interaction of Arrb2 with ERK1/2 and Akt was also reported previously in other cell types such as endothelial cells and neural progenitor cells; however, the present study was the first to report this interaction in EPCs 22, 40.

The proangiogenic properties of Arrb2 observed *in vitro* in EPCs might be of interest for improving cell-based therapeutics. Although the therapeutic potential of EPCs in treating ischemic vascular disease seemed promising, the extremely low number and functional impairment of EPCs associated with older age or other cardiovascular risk factors hindered their application 14, 41. To overcome this limitation, gene modification was proposed as a strategy to achieve the phenotypic modulation of EPCs leading to enhanced angiogenic properties 20, 42. The results of the present study showed that the transplantation of EPCs overexpressing Arrb2 significantly enhanced new vessel formation, improving better limb perfusion in the mouse ischemic hind-limb model. Animals receiving EPCs lentivirally transfected to knock down Arrb2 displayed a worse tolerance to ischemia. These results suggested that the clinical impact of the transplantation of EPCs modulated with Arrb2 might be significant compared with naive EPCs used in clinical trials.

This study had several limitations. First, although this study showed that PP2A was unlikely involved in Arrb2-mediated phosphorylation of Akt in EPCs, other mechanisms underlying the effect of Arrb2 on EPCs, besides ERK and Akt signaling pathways, need to be explored. For example, Laban et al. reported that Arrb2 recruited vasodilator stimulated phosphoprotein (VASP) in leukocytes to affect vascular repair after ischemia. Whether Arrb2 controls VASP activity in EPCs requires future investigation. Second, endothelial cells were also associated with ischemia-induced neovascularization, and this study showed that Arrb2 could improve endothelial cells migration, consistent with previous findings. However, Ma et al. demonstrated the role of Arrb1 in promoting endothelial cells function through VEGFR3. The present study found that Arrb2, but not Arrb1, was responsible for the EPC behavior. Similarly, Wang et al. also demonstrated that Arrb2, instead of Arrb1, was important in the mediation of cardiac ischemia-reperfusion injury. The functional divergence of Arrb1 and Arrb2 might be attributed to their different subcellular distributions and distinct interacting proteins in different cell types. These possibilities need further exploration.

In conclusion, the findings of this study clearly suggested a critical role for Arrb2 in vascularization via increasing the number of EPCs and improving their functions through the activation of ERK and Akt pathways. This novel biological function of Arrb2 might provide a potential therapeutic option to modulate EPCs in the treatment of ischemic vascular diseases.

## Supplementary Material

Supplementary figuress.

## Abbreviations

Akt: protein kinase B
Arrb2: β-arrestin 2
EPCs: endothelial progenitor cells
ERK: extracellular signal-regulated kinases
p-Akt: phosphorylated protein kinase B
p-ERK: phosphorylated extracellular signal-regulated kinases
