# Supplementary material for: Arrb2 promotes endothelial progenitor cell-mediated postischemic neovascularization
Source: Theranostics. 2020 Aug 6;10(21):9899–912. doi: 10.7150/thno.45133 (PMC7449919; doi:10.7150/thno.45133)
Supplement: Supplementary file 1 — Supplementary figuress. [file thnov10p9899s1.pdf]

Arrb2 promotes endothelial progenitor cell-mediated postischemic neovascularization

Supplemental Figures and Figure Legends:

Figure S1: Identification of EPCs from human umbilical cord blood.

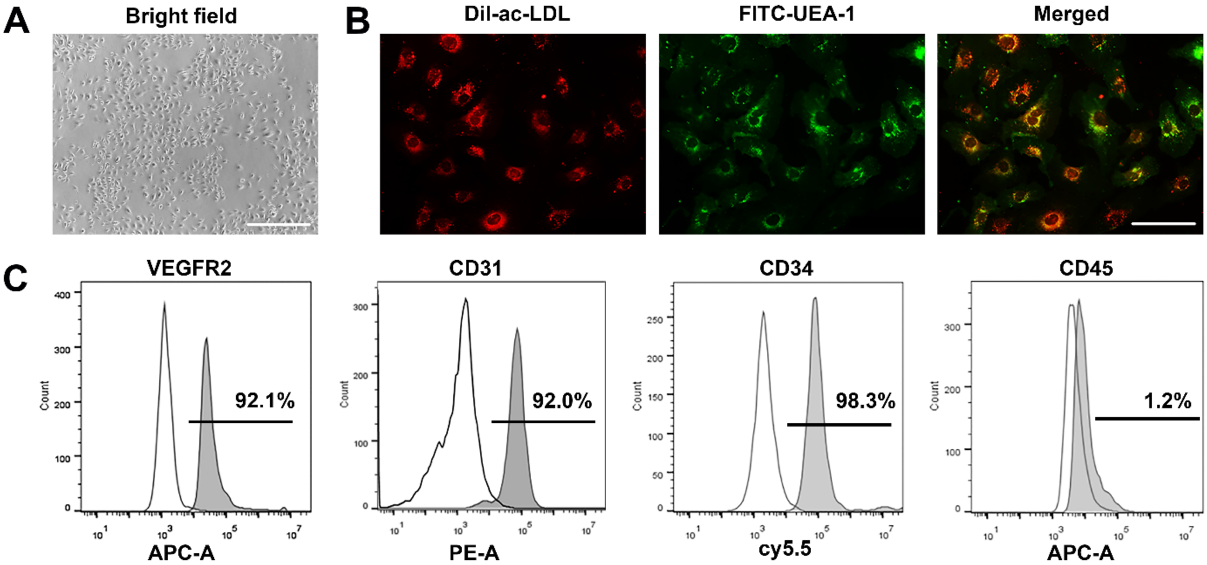

Figure S1: A) After 15-day culture of mononuclear cells, spindle-shaped or cobblestone-like adherent cells were observed in bright field. Scale bar=400 µm. **B)** Representative images of Dil-ac-LDL and FITC-UEA-1 staining showing these cells were able to take up Dil-ac-LDL and bind FITC-UEA-1. Scale bar=100 µm. **C)** Representative images of flow cytometry showing these cells expressing progenitor cell–specific surface antigens CD34 and endothelial cell–specific surface antigen VEGFR2 and CD31.

Figure S2: The expression of Arrb1 and Arrb2 in EPCs after hind-limb ischemia induction or hypoxia treatment.

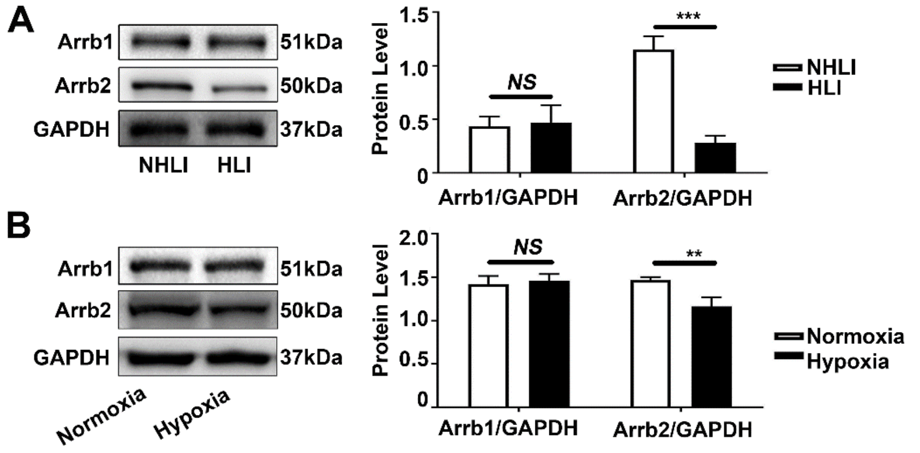

Figure S2: A) The protein expression of Arrb2 was profoundly reduced compared with non-ischemia group. However, Arrb1 expression was not altered. **B)** Arrb2 expression was overtly decreased in EPCs after hypoxia treatment, but hypoxia injury didn't change the protein level of Arrb1 in EPCs. All data are expressed as mean±SD (n=3), $^{**}p<0.01$, $^{***}p<0.001$ compared with control.

Figure S3: The effectiveness of Lv-Arrb2 and sh-Arrb2 in EPCs was confirmed.

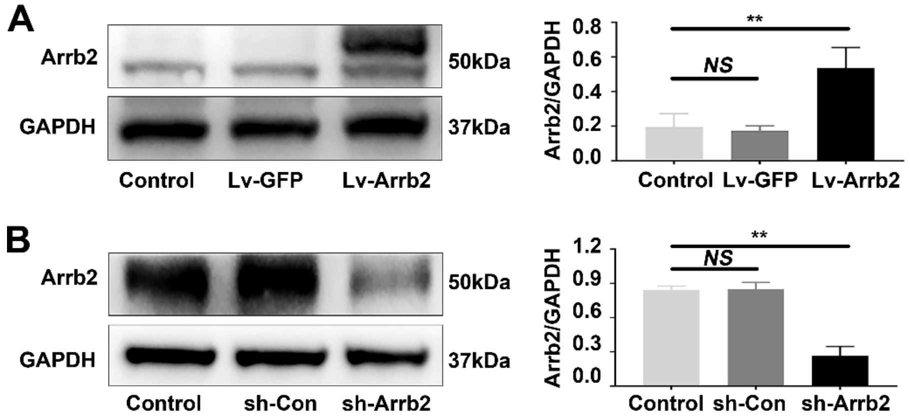

Figure S3: A) Effect of Lv-Arrb2 on Arrb2 expression was determined by Western blot analysis and then quantitated by densitometric analysis. **B)** Effect of sh-Arrb2 on Arrb2 expression was determined by Western blot analysis and then quantitated by densitometric analysis. Values are means±SD (n=3), **$p<0.01$ compared with control.

Figure S4: Arrb2 improves endothelial cells migration

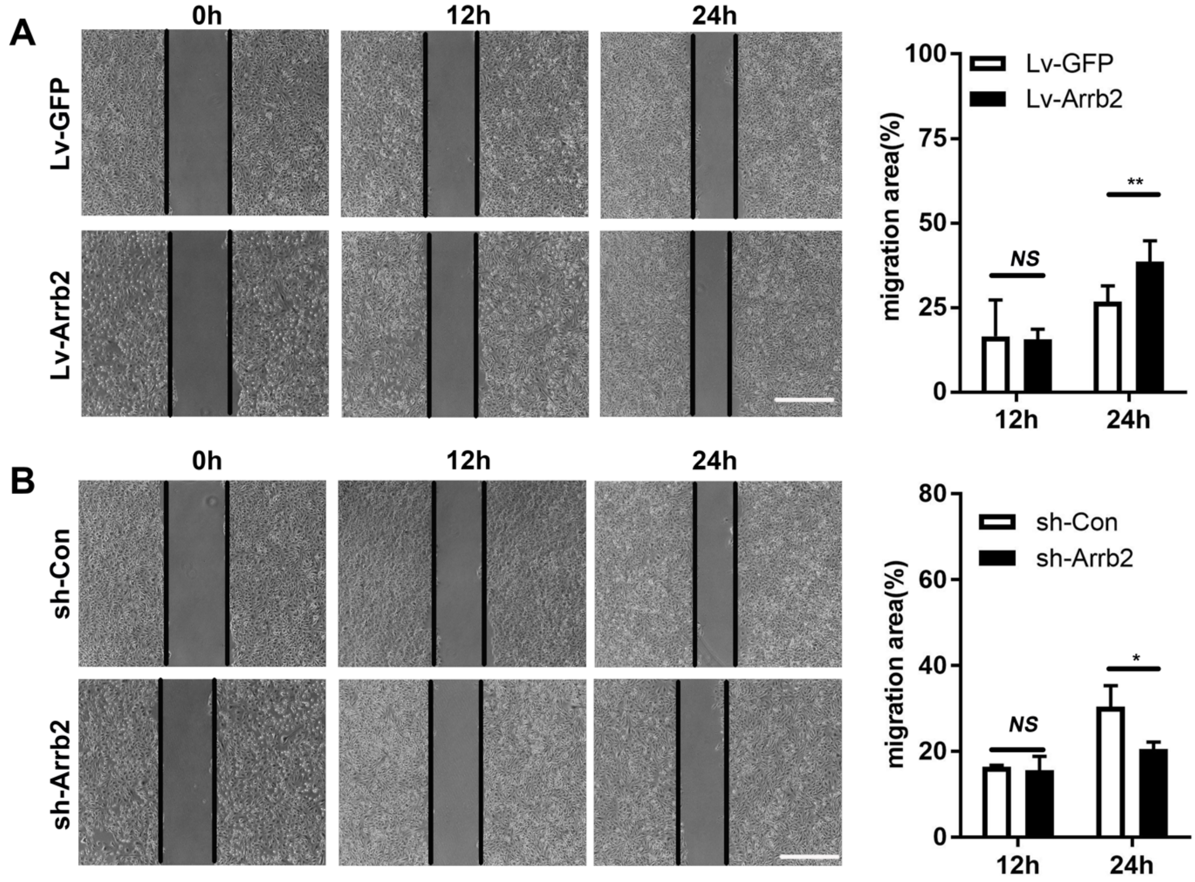

Figure S4: A) endothelial cells were infected with Lv-Arrb2 or Lv-GFP for 48 h. Representative images of the in vitro scratch-wound assay and quantification of the migration area are presented to show the effects of Lv-Arrb2 on cell migration. Scale bar=400 µm. **B)** endothelial cells were infected with sh-Arrb2 or sh-Con for 48 h. Representative images of the in vitro scratch-wound assay and quantification of the migration area are presented to show the effects of sh-Arrb2 on cell migration. Scale bar=400 µm. All data are expressed as mean±SD (n=5), *p<0.05, **p<0.01, ***p<0.001 compared with control.

Figure S5: The effect of sh-Arrb2 and ERK1/2 inhibitor on the proliferation, migration and angiogenic function of EPCs.

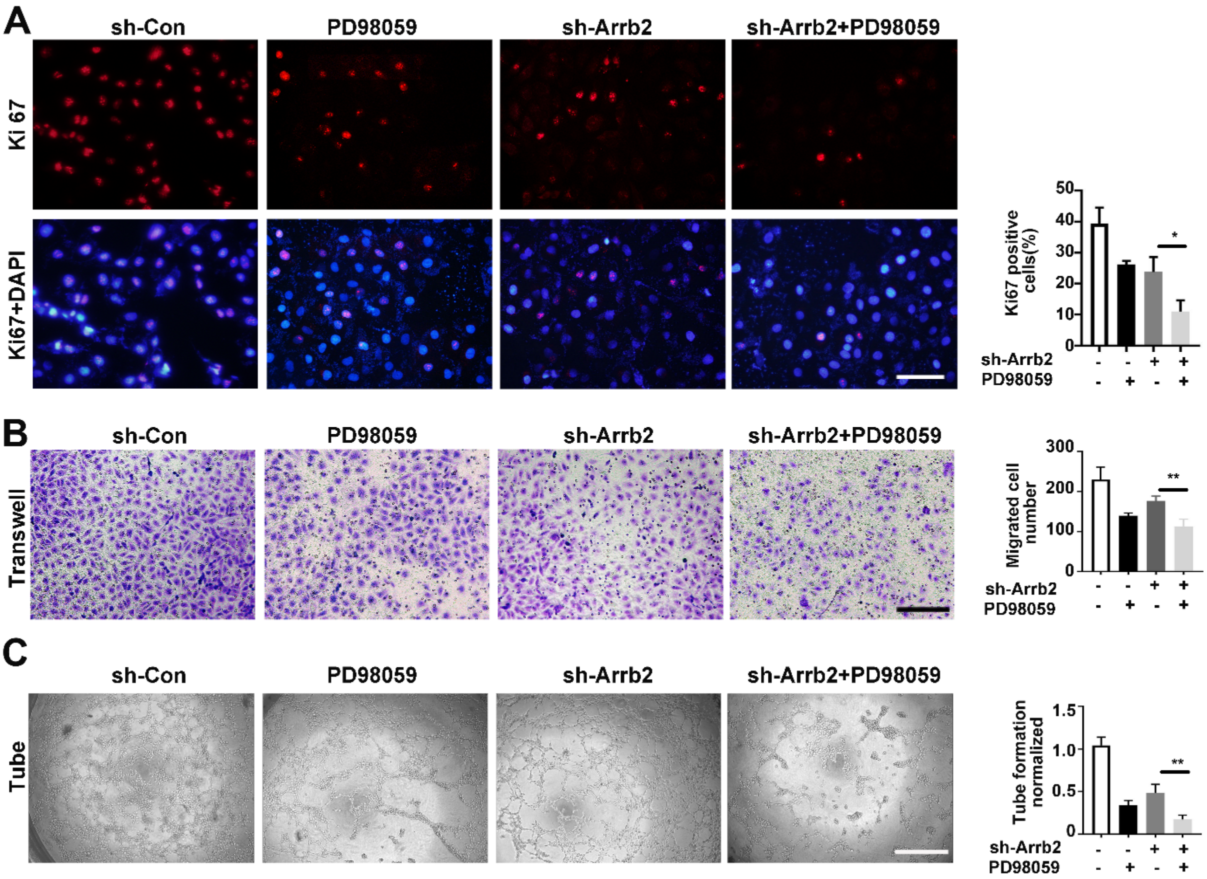

Figure S5: A) Representative images and quantification of Ki67 staining showing the decrease of proliferation in Arrb2-knockdown EPCs was aggravated by ERK1/2 inhibitor (PD98059). Scale bar=100 μm. **B)** Representative images of Transwell migration assay and quantification of the migrated cells showing the decrease of migration in Arrb2-knockdown EPCs was aggravated by ERK1/2 inhibitor (PD98059). Scale bar=200 μm. **C)** Representative images and quantification of tube formation showing the decrease of tube formation in Arrb2-knockdown EPCs was aggravated by ERK1/2 inhibitor (PD98059). Scale bar=400 μm. All data are expressed as mean±SD (n=5). *p<0.05, **p<0.01compared with sh-Arrb2.

Figure S6: The effect of sh-Arrb2 and Akt inhibitor on the proliferation, migration and angiogenic function of EPCs.

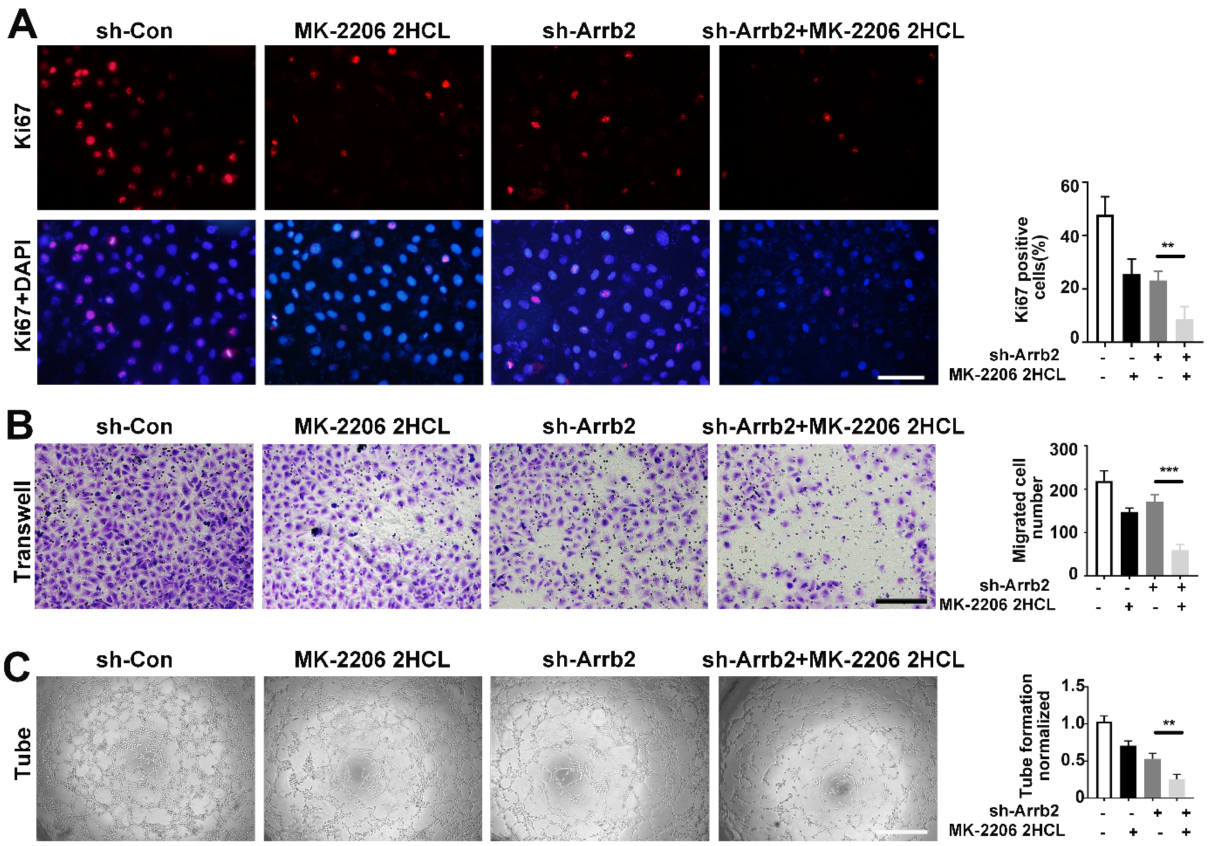

Figure S6: A) Representative images and quantification of Ki67 staining showing the decrease of proliferation in Arrb2-knockdown EPCs was aggravated by Akt inhibitor (MK-2206 2HCL). Scale bar=100 μm. **B)** Representative images of Transwell migration assay and quantification of the migrated cells showing the decrease of migration in Arrb2-knockdown EPCs was aggravated by Akt inhibitor (MK-2206 2HCL). Scale bar=200 μm. **C)** Representative images and quantification of tube formation showing the decrease of tube formation in Arrb2-knockdown EPCs was aggravated by Akt inhibitor (MK-2206 2HCL). Scale bar=400 μm. All data are expressed as mean±SD (n=5). **p<0.01, ***p<0.001 compared with sh-Arrb2.

Figure S7: The expression of Arrb2 in EPCs from Arrb2$^{-/-}$ mice and wild type mice.

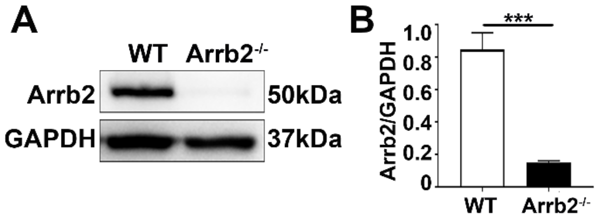

Figure S7: The expression of Arrb2 in EPCs isolated from Arrb2$^{-/-}$ mice and wild type (WT) mice was determined by Western blot analysis and then quantitated by densitometric analysis Data are expressed as mean±SD (n=3). ***p<0.001 compared with WT.
